# Chiral Alanine Functionalized Perylene Diimides: Supramolecular Self-Assembly and Optical Properties

**DOI:** 10.3390/molecules31173102

**Published:** 2026-09-04

**Authors:** Di Fan, Fan Qi, Nan Li, Rongli Liu, Haitao Ren, Guodong Fan

**Affiliations:** 1 School of Artificial Intelligence, Shaanxi Polytechnic University, Xianyang 712000, China; 2 School of Medicine, Xi’an Peihua University, Xi’an 710125, China; 3 College of Chemistry and Materials Science, Weinan Normal University, Weinan 714099, China; 4 Key Laboratory of Auxiliary Chemistry &Technology for Chemical Industry, Shaanxi University of Science & Technology, Ministry of Education, Xi’an 710021, China; 5 Shaanxi Key Laboratory of Liquid Crystal Polymer Intelligent Display, Technological Institute of Materials & Energy Science (TIMES), Xijing University, Xi’an 710123, China

**Keywords:** PDI, alanine, photoluminescence, aggregation, self-assembly

## Abstract

Water-soluble alanine-functionalized perylene diimide derivatives (PDI-Ala) were synthesized by introducing alanine units at the imide positions of the PDI framework. Their photoluminescence properties and supramolecular aggregation behavior were investigated in solvents of varying polarity. Chiral L-PDI-Ala displayed distinct solvent-dependent emission colors and intensities, whereas the achiral PDI-Ala showed negligible emission under the same conditions. Poor solvents such as acetone and ethyl acetate promoted relatively efficient emission with hypsochromically shifted bands, while good solvents such as N, N-dimethylformamide and dimethyl sulfoxide induced bathochromically shifted emission with much lower quantum yields. The results indicate that strong face-to-face π-π stacking in the solid-state leads to aggregation-caused quenching (ACQ), whereas ordered aggregates formed by intermolecular forces such as hydrogen bonds or stacking in solution can recover luminescence. On the basis of the spectroscopic and structural results, a solvent-regulated emission mechanism involving excimer/exciplex formation together with H-type and J-type aggregation is proposed. This work provides useful insight into the aggregation-controlled optical behavior of chiral amino-acid-modified PDI materials and offers guidance for the design of chiroptical and self-assembled luminescent systems.

## 1. Introduction

Perylene diimide (PDI) has attracted extensive interest in organic electronics because of its excellent chemical robustness, high photostability, strong electron-accepting ability, and versatile synthetic tunability [1]. However, the rigid planar aromatic core of PDI usually drives strong intermolecular π-π stacking in the solid state and in aqueous media, which commonly causes aggregation-caused quenching (ACQ) [2,3]. In addition, the intrinsically poor water solubility of PDI greatly limits its applications in solar cells, displays, fluorescent sensing, bioimaging, and phototheranostics [4,5].

Current modification strategies for PDI primarily focus on the imide position [6,7,8], bay [9,10], and the ortho position [1]. Among these three approaches, the synthetic routes for bay- and ortho-position modifications are relatively complex and often require harsh reaction conditions. For bay-position substitution, the introduction of bulky groups can induce twisting of the PDI plane [7,11]. Introducing electron-withdrawing or electron-donating groups at the bay position significantly alters the electronic and optical properties of PDI. For instance, the introduction of electron-donating groups can lead to a red shift in the absorption/emission spectra, accompanied by improvements in fluorescence quantum yield and conductivity [9]. Substitution at the ortho position can largely preserve the planarity of the perylene core but mainly influences the solubility and molecular packing of the entire molecule through the steric hindrance and hydrophilicity/hydrophobicity of the substituents [12]. In contrast, modification at the imide position, typically by introducing hydrophilic groups, is widely adopted due to its synthetic simplicity, ease of reaction control, and straightforward product purification [13]. This strategy effectively enhances the dye’s aqueous solubility. Moreover, because the imide position imposes minimal steric hindrance, the planar architecture of the PDI core is largely preserved. The resulting extended conjugated framework retains the strong self-assembly propensity characteristic of pristine PDI [14,15] while exerting negligible influence on its optical properties. Nevertheless, the major limitation of this approach remains the insufficient water solubility, along with the difficulty of introducing chiral helical structures through molecular twisting via this synthetic route.

Currently, strategies for constructing chiral PDIs can be categorized into three types: twisting the planar core, introducing chiral substituents, and co-assembly with chiral guest molecules [16]. (1) Twisting the planar core is commonly achieved by introducing bulky substituents at the bay [17] or ortho positions of PDI, or by fusing helicenes via ring closure at bay/ortho positions. These methods induce twisting of the perylene aromatic core along its long or short axis, forming chiral structures such as bowls, helices, or ribbons [1]. However, modifications at the bay or ortho positions typically involve multi-step organic syntheses under harsh reaction conditions (e.g., requiring precious metal catalysts and inert gas protection). These processes often suffer from low yields and numerous by-products, increasing purification difficulty and cost [18,19,20]. Furthermore, precise control over the degree of plane twisting is challenging. The charge transfer (CT) effect between PDI and introduced electron-donating groups, π-π stacking modes, molecular symmetry and rigidity, and solvent polarity can all significantly influence the directionality of helix (or layer-stacking) formation and the degree of twist (rotation), thereby markedly affecting the intensity of circular dichroism (CD) signals [14]. Excessive twisting of the plane can even disrupt the original planar conjugated structure of PDI, damage its chiral helical architecture, and lead to the disappearance of chiral luminescence. The introduced steric hindrance or electronic effects may also impact the structural, chemical, and thermal stability of the molecule, complicating the control over aggregation behavior. (2) Chiral Substituents: A more effective and straightforward method for constructing chiral molecules involves modifying the imide positions of PDI with different chiral substituents. Through controllable self-assembly and chiral transmission, this approach allows for the construction of chiral PDI supramolecular structures [21,22]. This method facilitates rapid, multi-level chiral transfer from individual molecular units to macroscopic helical aggregates [23,24]. Commonly used chiral substituents are natural chiral molecules [15], such as amino acids, peptides, and sugars, or synthetic chiral molecules like chiral anilines and chiral alkylamines. Marius Wehner et al. [25] systematically studied the self-assembly behavior of a racemic mixture of (R,R)- and (S,S)-PBI derived from PDI modified with chiral amino acids. Their research found that homochiral aggregation of the racemic mixture leads to the formation of two types of supramolecular conglomerates under kinetic control, while under thermodynamic control heterochiral aggregation is preferred, affording a racemic supramolecular polymer. Xiaobo Shang et al. [26] synthesized an imide-substituted N,N’-bis-(1’-phenylethyl) perylene-3,4,9,10-tetracarboxyldiimide using chiral aniline as the chiral source. Their study found that heterochiral nanowires were formed by the co-self-assembly of the distinct enantiomers. Interestingly, these heterochiral nanowires consisted of alternating sequences of the opposite enantiomers. On the other hand, homochiral nanowires obtained from a pure enantiomeric system exhibited the highest electron mobility among chiral organic semiconductors, surpassing the hole mobility of p-channel organic chiral materials by two to three orders of magnitude. Furthermore, their supramolecular chirality and enhanced photoresponsivity enabled circularly polarized luminescence detection with higher selectivity compared to their thin films. Based on the cogwheel model of hierarchical self-organization, Li Wang et al. [27] discovered a method for rapid chiral self-assembly. The chirality in this system originated from aromatic groups bearing three alkoxy chains at the imide position. The driving force for the hierarchical self-organization was attributed to crystal packing and local chain dynamics. The condition for the cogwheel model of self-organization is matching the chain length to the half-pitch length of the helix. Ke-Rang Wang et al. [28] prepared a lactose-functionalized perylene bisimide (PBI-Lac) using 2-aminoethylhepta-O-acetyl-b-D-lactoside as the chiral precursor. In a DMSO-H_2_O mixed solvent, it self-organized into a right-handed supramolecular structure. Benefiting from the grafting of D-lactose, the self-assembly of PBI-Lac as a multivalent glycocluster exhibited specific binding with peanut agglutinin. With regard to chiral substituent modification, incorporating chiral amino acids at the imide positions preserves the inherent π-π stacking ability of the PDI framework. Upon dispersion in suitable solvents, the entropy of dissolution drives these planar PDI derivatives bearing chiral substituents to self-assemble into helically arranged H-type aggregates centered around the π-π plane, affording architectures with maximized chiral luminescence [16]. Nevertheless, relevant investigations pertaining to this subject remain limited. (3) Co-assembly with Chiral Guest Molecules: This approach involves constructing chiral PDI supramolecular assemblies by transferring chirality from chiral guest molecules to achiral PDIs through co-assembly. In this strategy, chiral guest molecules typically act as donors, while achiral PDIs serve as acceptors. Driven by non-covalent weak interactions, such as hydrogen bonding, *π*-*π* stacking, hydrophobic interactions, and electrostatic forces [29,30,31,32], they co-assemble into chiral macromolecular structures. Unfortunately, the currently available pool of suitable chiral source molecules for such co-assembly is limited, and the efficiency of chirality transfer remains relatively low [14,33].

Previous reports on amino-acid-modified PDI derivatives have primarily focused on the detection of anions in single solvents [6] or the determination of natural antibiotics in aqueous media [8]. However, investigations into their luminescent properties have been largely confined to single or mixed solvent systems [7], and a systematic study of their concentration-dependent luminescent behavior across different solvents remains scarce. Fei Li et al. [7] reported D/L-alanine-modified PDI enantiomers and investigated their concentration-dependent aggregation behavior in chloroform/methanol mixed solvents, revealing a fluorescence red-shift and the emergence of circularly polarized luminescence signals upon the transition from monomer to aggregate states. While this work systematically demonstrated the influence of concentration on luminescent properties within a specific binary solvent system, it did not extend to a broader range of solvent types. Cordula D. Schmidt et al. [34] studied water-soluble PDIs modified with lysine and alanine and found that the formation of chiral supramolecular structures was concentration-dependent. Geraldine Echue et al. [35], using phenylalanine derivatives as building blocks, investigated the concentration-dependent self-assembly behavior in aqueous solution. These studies, however, were all confined to specific solvents (water or buffer solutions) and lacked a systematic comparison of concentration effects across different solvents. Therefore, a comprehensive investigation in this direction is not only conducive to a deeper understanding of the physicochemical origin of supramolecular chirality generation and transfer in PDIs but also provides theoretical guidance for the development of solvent-tunable intelligent optical materials.

Based on the above discussion and given the poor water solubility of PDI-based electronic materials, we modified PDI by introducing chiral alanine, which bears a highly water-soluble structure, at the imide position. The successful synthesis of the target compound was confirmed by ^1^H NMR and ^13^C NMR spectroscopy. Furthermore, a combination of optical, structural, and morphological methods was employed to systematically investigate the photoluminescence properties, aggregation behavior in solvents of different polarities, and the underlying photoluminescence mechanism of the PDI-Ala derivative. These findings provide important theoretical and experimental references for the design and preparation of amino acid-functionalized chiral PDI derivative luminescent materials.

## 2. Results and Discussion

### 2.1. Analysis of Luminescence Behavior

**Figure 1:**
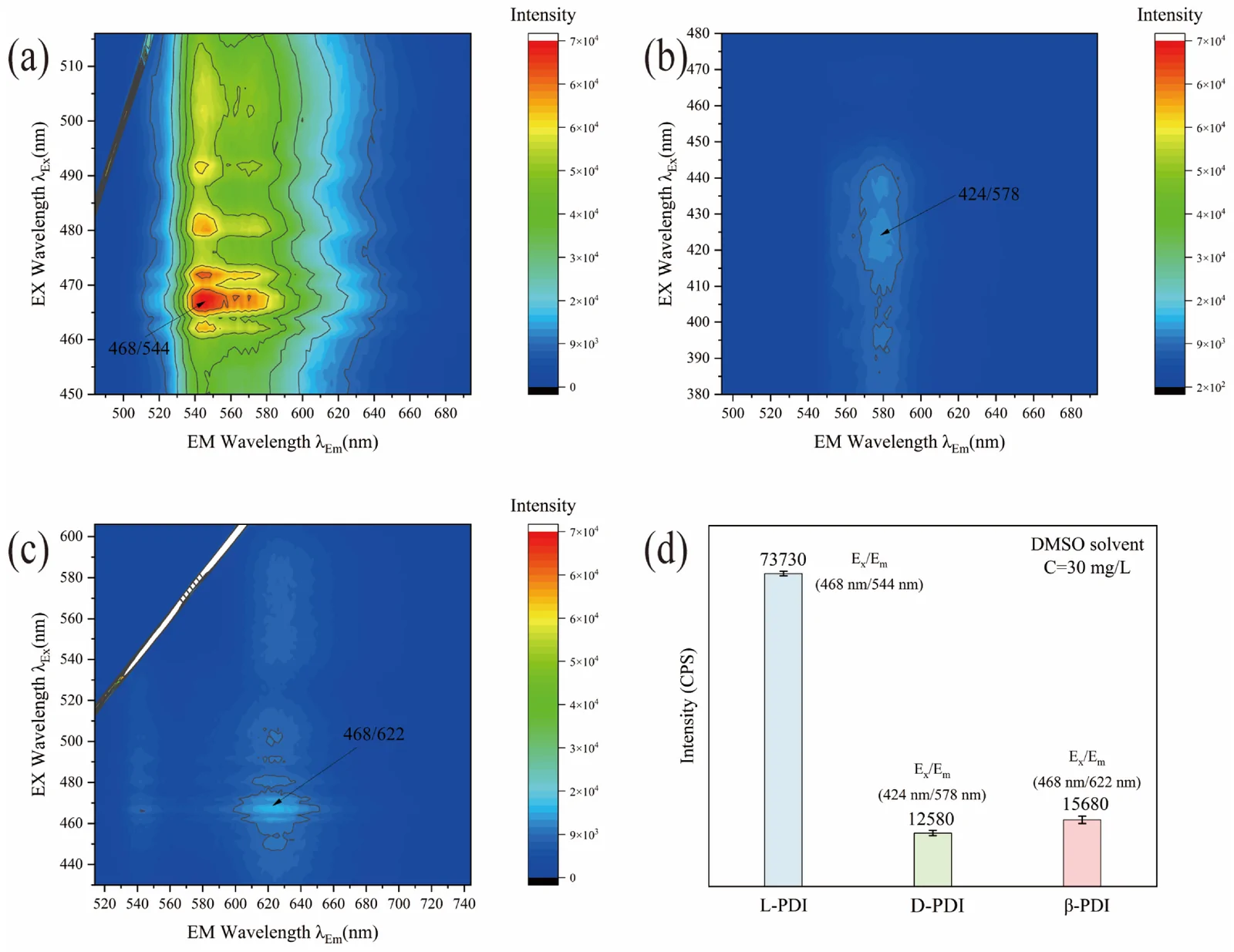
3DEEM fluorescence spectra of PDI dissolved in DMSO. (**a**) L-PDI, (**b**) D-PDI, (**c**) β-PDI, (**d**) Bar chart showing the PL intensities of PDI-Ala.

Taking DMSO as a solvent, the concentration of all samples was fixed at 30 mg/L. The luminescent behaviors of L-PDI, D-PDI, and β-PDI were interrogated by 3DEEM spectroscopy, and the results are compiled in Figure 1. As shown, the brightest emission maxima for LPDI, D-PDI, and β-PDI are located at Ex/Em = 468/544 nm, 424/578 nm, and 468/622 nm, respectively, manifesting a progressive bathochromic shift in the maximum emission wavelength. Figure 1d compares the emission intensities at these peak positions. In DMSO, L-PDI exhibits markedly stronger emission than both D-PDI and β-PDI, indicating that among the three structurally differentiated PDI derivatives, L-PDI possesses the highest emission efficiency. Consequently, L-PDI was selected as the model compound for subsequent luminescence investigations.

Introduction of water-soluble alanine into the PDI backbone led to markedly enhanced aqueous solubility for the resulting PDI-Ala derivatives, with L-PDI exhibiting a wine-red color in water. Nevertheless, negligible or only extremely weak luminescence was observed both in the solid state and when dissolved in water, which can be ascribed to the essentially unchanged planar geometry of the perylene core following imide-position functionalization. Consequently, intermolecular aggregation in the solid state and in water remains predominantly governed by face-to-face π-π stacking (H-aggregates).

**Figure 2:**
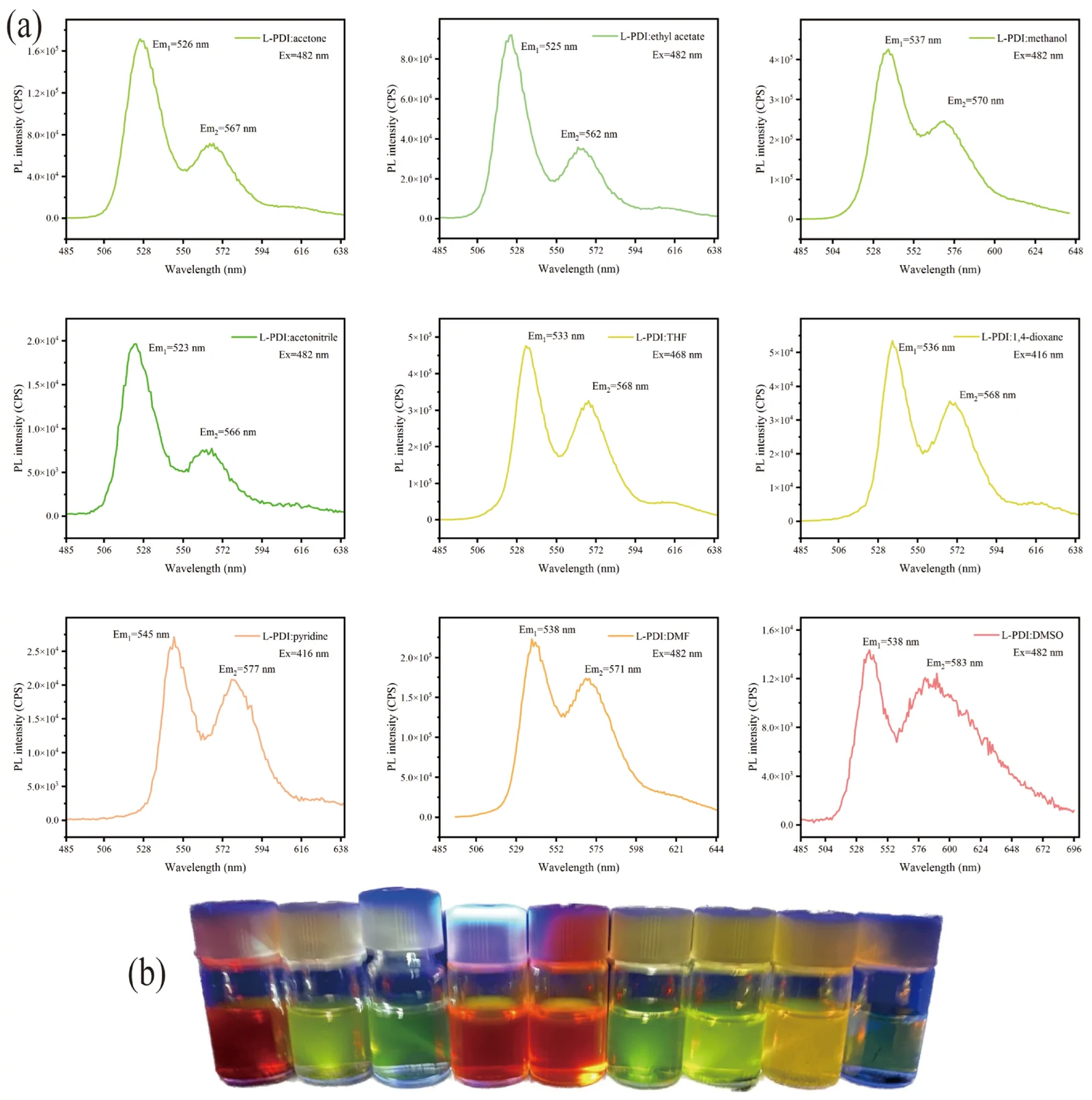
(**a**) PL spectra and (**b**) Photograph of PL for L-PDI in different polar solvents. The solvents from left to right are DMSO, methanol, acetonitrile, DMF, pyridine, acetone, 1,4-dioxane, THF and ethyl acetate, concentration is 10 mg/L.

L-PDI was dissolved in a range of solvents with progressively varying polarity. Notably, the resulting solutions displayed solvent-dependent emission colors, indicating that the PL properties of L-PDI are sensitive to the surrounding solvent environment. As shown in the photograph in Figure 2b, L-PDI exhibited variable PL colors across different solvents. This color variability arises from the formation of distinct aggregates by chiral L-PDI molecules in solvents of different polarities [33,36,37], where each aggregate type possesses different energy levels, leading to fluorescence emission at different wavelengths. Figure 2a presents the PL spectra of L-PDI in nine different solvents. Table 1 summarizes the corresponding excitation wavelengths, main emission wavelengths, and PLQY in different solvents. Under identical concentration conditions, the order of emission intensity (integrated area) for L-PDI across solvents (from strongest to weakest) is as follows:

THF (2.5169 × 10^7^) > Methanol (2.0517 × 10^7^) > DMF (1.1446 × 10^7^) > Acetonitrile (7.4665 × 10^6^) > Acetone (6.6136 × 10^6^) > Ethyl acetate (3.1667 × 10^6^) >1,4-Dioxane (2.2628 × 10^6^) > DMSO (1.2941 × 10^6^) > Pyridine (1.2821 × 10^6^).

The results in Figure 2a and Table 1 show that the emission spectra in all solvents exhibit a double-peak profile, with the shorter-wavelength peak being more intense than the longer-wavelength one. Figure S1 and Table S1 show the CIE chromaticity coordinates of L-PDI emission in the aforementioned solvents.

To further investigate the photoluminescence efficiency of L-PDI in different solvents, the PLQYs of different solutions were measured using an integrating sphere, and the test results are presented in Table 1 and Figure S2. Comparative analysis revealed that solvents with higher PLQYs, acetone, ethyl acetate, methanol, acetonitrile, THF, and 1,4-dioxane, show their shorter wavelength emission maxima in the range of 523–537 nm and their longer wavelength maxima in the range of 562–568 nm, collectively appearing as green and yellow fluorescence. In contrast, solvents with lower PLQYs, pyridine, DMF, and DMSO, exhibit shorter wavelength maxima between 538 and 545 nm and longer wavelength maxima between 571 and 590 nm, appearing as red-orange fluorescence.

The luminescence behavior in solvents of different polarities is complex and is influenced by multiple factors, including the solvent’s dielectric constant, polarity index, solubility, and the number of hydrogen bonding acceptors and donors present in the solvent molecule. Table 2 lists the relevant parameters for each solvent. It can be inferred that solvents that are relatively poor solvents for L-PDI and which contain more hydrogen bonding acceptors/donors and more terminal methyl groups tend to yield higher PLQYs. A plausible explanation is that these solvents can engage in intermolecular hydrogen bonding with L-PDI or facilitate π-π stacking and *σ*-H···π interactions within the aggregates.

**Figure 3:**
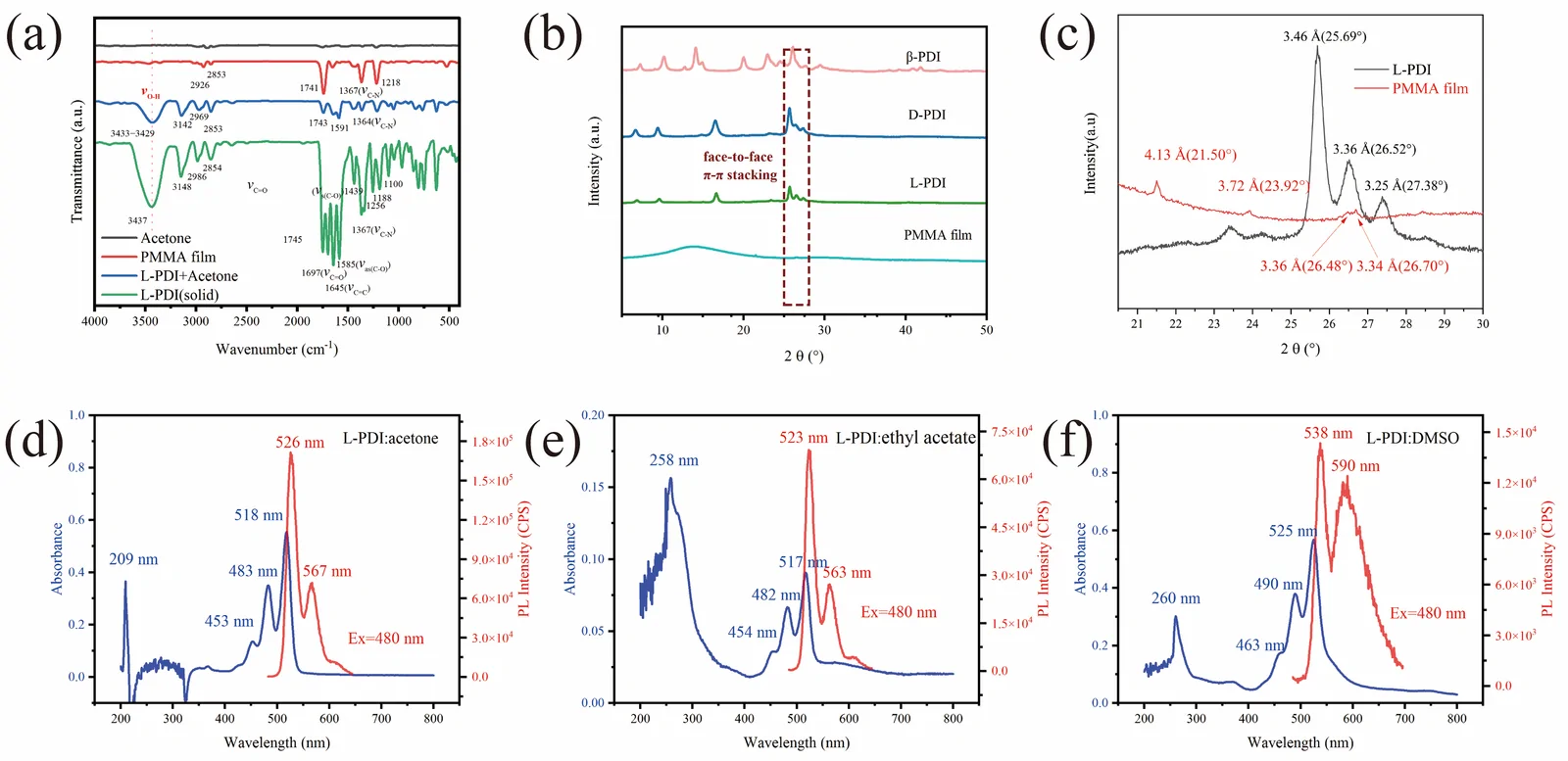
(**a**) FT-IR spectra of L-PDI under different states: neat solid, solid with residual acetone, and PMMA composite film cast from acetone solution, (**b**,**c**) XRD patterns of PDI-Ala and PMMA composite film, (**d**–**f**) UV-Vis absorption and PL spectra of L-PDI in poor and good solvents, solution concentration is 10 mg/L.

To investigate the difference in the PL behavior of L-PDI in the solid state versus in acetone solution, the luminescent acetone solution of L-PDI was solidified within a polymethyl methacrylate (PMMA) film. Figure 3a shows the FT-IR spectra of solid L-PDI, L-PDI with partially evaporated acetone, and L-PDI acetone solution solidified in a PMMA film. In the spectrum of solid L-PDI, a broad and strong peak appears at approximately 3437 cm^−1^. This peak corresponds to the O–H stretching vibration of the carboxyl group. The wavenumber is lower than 3500 cm^−1^, which serves as evidence for the presence of hydrogen bonding between L-PDI molecules in the solid state. When a small amount of acetone is added (L-PDI + Acetone), the characteristic hydroxyl peak of the compound shifts slightly to a lower wavenumber, appearing as a broad, strong absorption band centered around 3429–3433 cm^−1^. This indicates that a significant number of associated hydrogen bonds still exist between L-PDI molecules in acetone. At this point, the aggregated L-PDI still showed no observable photoluminescence, suggesting that strong face-to-face π-π stacking persists among a large number of L-PDI molecules, leading to ACQ. However, dilute solutions of L-PDI exhibited intense PL. When the luminescent acetone solution was solidified in a PMMA film, the resulting film containing L-PDI molecules still showed strong PL. Notably, the characteristic absorption peak of associated hydrogen bonds was almost undetectable in the FT-IR spectrum of the luminescent PMMA film. This is because the solvent acetone had completely evaporated, and the luminescence behavior of L-PDI immobilized in the PMMA film is governed by the intermolecular packing mode.

Figure 3b shows the XRD patterns of PDI-Ala. All PDI-Ala compounds in the solid state exhibited strong characteristic diffraction peaks corresponding to face-to-face π-π stacking, indicating that imide-position modification of PDI largely preserves the planar conjugated structure of the perylene aromatic core. Figure S3 and Table S2 provide the intermolecular d-spacings of all PDI-Ala in the solid state and in PMMA films, and the corresponding packing modes are marked. Figure 3c compares the stacking of L-PDI in the solid state and in acetone solution (solidified in a PMMA film). In the solid state, diffraction peaks at approximately 2θ = 27.38°, 26.52°, and 25.69° correspond to d-spacings of 3.25 Å, 3.36 Å, and 3.46 Å, respectively. These d-spacings represent the interplanar distances between the perylene cores, confirming that the molecules adopt a sandwich-like aggregated conformation with face-to-face stacking, also known as H-aggregation. The appearance of three closely spaced diffraction peaks is attributed to a certain degree of offset in π-*π* stacking caused by electrostatic repulsion between π-electron clouds. In contrast, L-PDI molecules “frozen” within the PMMA film exhibit d-spacings in a narrower range of 3.34–3.36 Å, suggesting that L-PDI molecules in acetone solution can form a more ordered H-aggregation. This structure represents a high-entropy kinetic assembly that can undergo conformational changes under suitable conditions to form a more stable thermodynamic structure, J-aggregation. It is noteworthy that two distinct diffraction peaks appear at lower 2θ angles: 2θ = 23.92° and 21.50°, corresponding to d-spacings of 3.72 Å and 4.13 Å, respectively. These peaks likely indicate a looser packing mode, possibly corresponding to loose dimers, excimers, or exciplexes.

Figure 3d–f compared the UV-Vis absorption and PL of L-PDI in poor solvents (acetone, ethyl acetate) and a good solvent (DMSO). It is evident that L-PDI in poor solvents shows a hypsochromic shift in both UV-Vis absorption and photoexcited emission wavelengths, while in a good solvent, both exhibit a bathochromic shift. The above observations can be rationalized as follows. DMSO, possessing a stronger hydrogen-bond-accepting ability than acetone and ethyl acetate, forms stronger intermolecular hydrogen bonds with the hydroxyl group of the carboxyl moiety in L-PDI. The dominant stabilization of the excited state over the ground state via these hydrogen-bonding interactions ultimately gives rise to a red shift in the emission of L-PDI in this good solvent.

### 2.2. Concentration, Chirality, and Temperature Dependence of the Photophysical Behavior of Alanine-Modified PDI Derivatives

**Figure 4:**
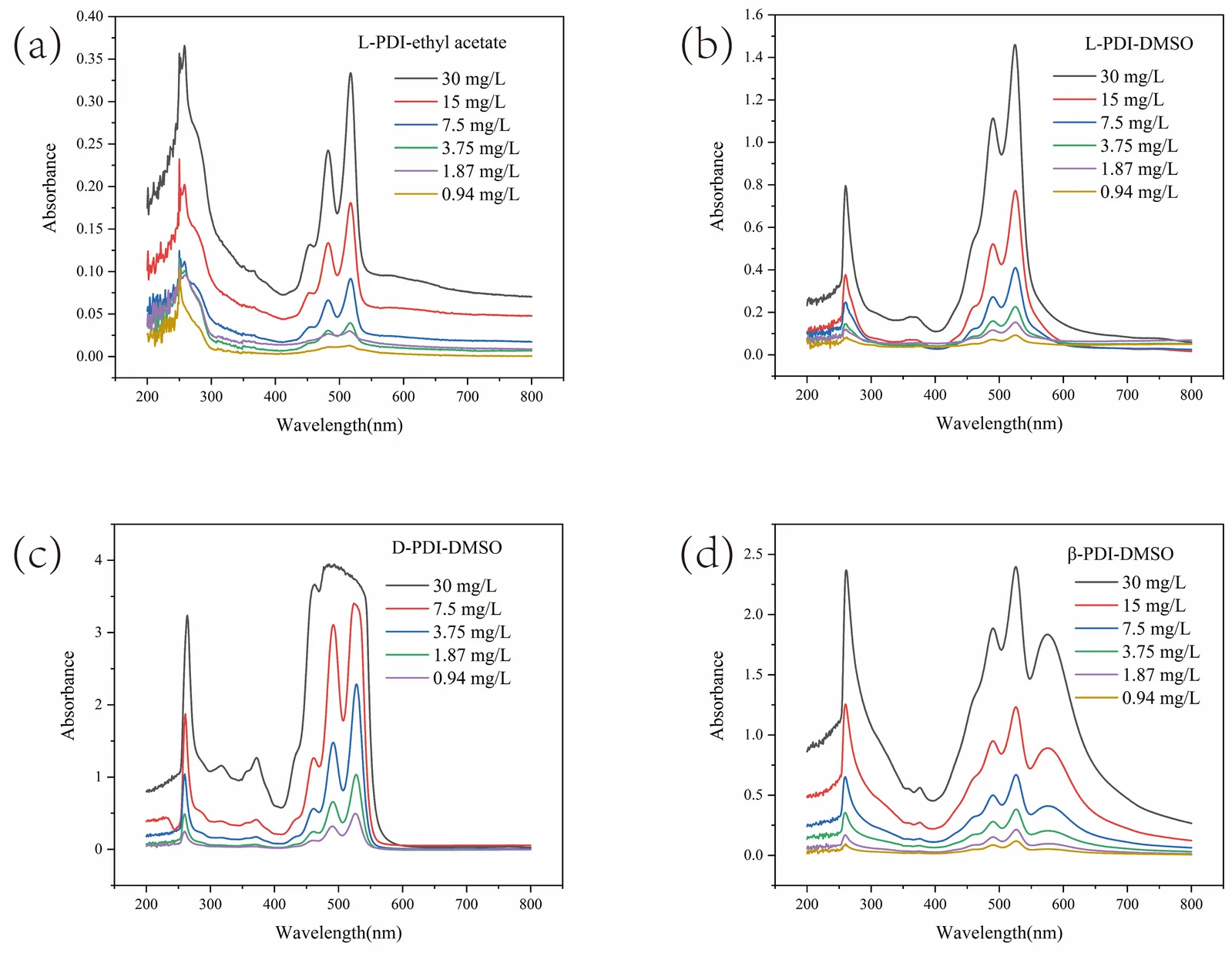
Concentration-dependent UV-Vis absorption spectra of PDI-Ala. (**a**) L-PDI in ethyl acetate, (**b**) L-PDI in DMSO, (**c**) D-PDI in DMSO, (**d**) β-PDI in DMSO.

To further investigate the concentration dependence, we selected ethyl acetate (poor solvent) and DMSO (good solvent) as two representative solvents and systematically examined the concentration-dependent UV-Vis absorption and PL spectra of L-PDI. Figure 4a shows the UV-Vis absorption spectra of three different PDIs dissolved in ethyl acetate and DMSO at varying concentrations. In the concentration range of 0.94–30 mg/L, with the exception of the D-PDI/DMSO solution at 30 mg/L, the three PDI derivatives in DMSO (Figure 4b,c) exhibit distinct differences in both spectral line shapes and the positions of the maximum absorption peaks (i.e., absorption wavelengths), which are attributable to structural differences among the PDI molecules. In contrast, the UV-Vis absorption spectra of L-PDI in ethyl acetate and DMSO (Figure 4a,b) display largely similar line shapes and maximum absorption peak positions, indicating that the solvent effect is less significant than the influence of the molecular structure itself. For a given compound-solvent system, the spectral line shape and the maximum absorption peak position remain essentially unchanged with concentration. Only the absorbance intensity varies with concentration.

**Figure 5:**
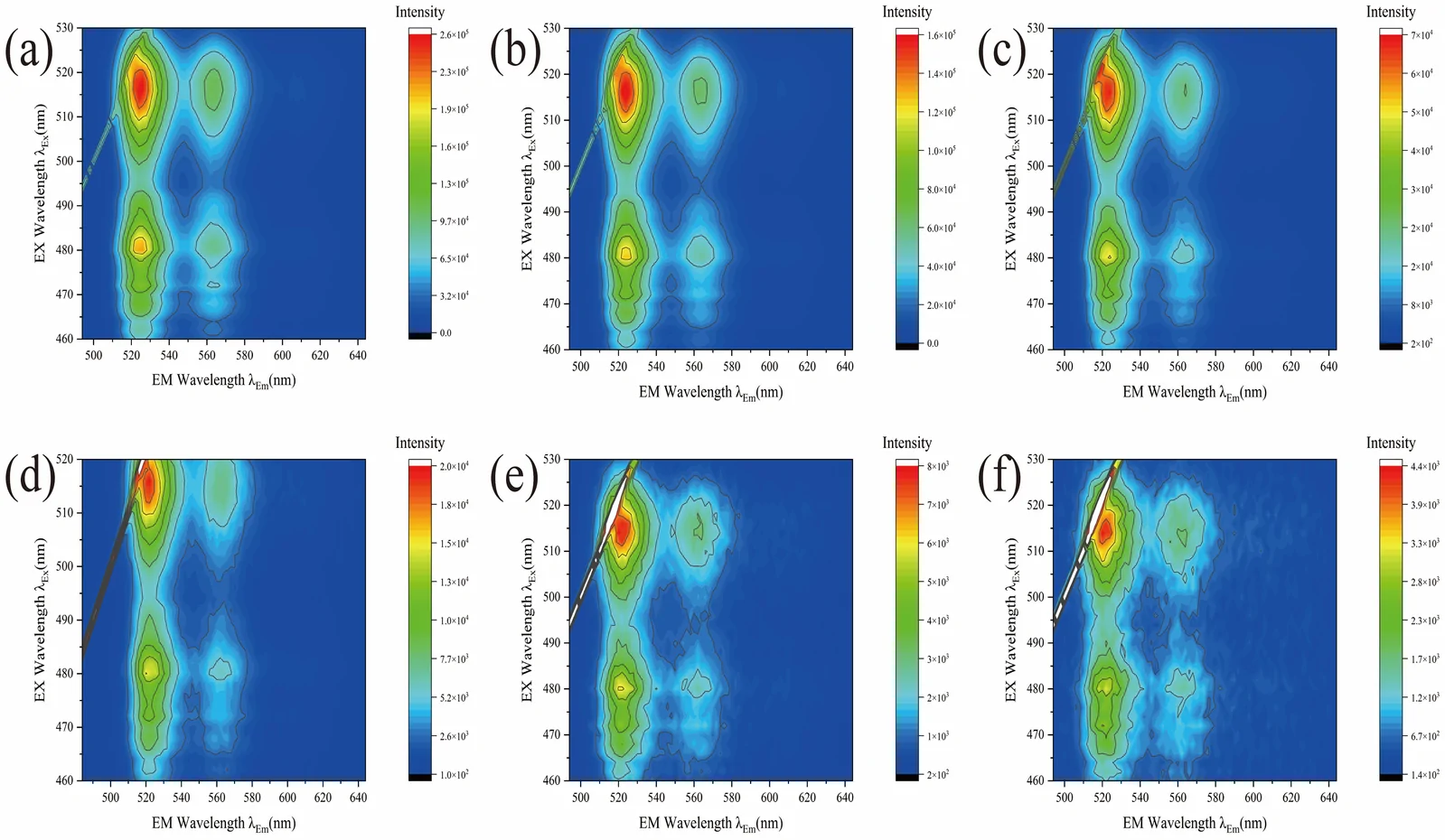
Concentration-dependent 3DEEM fluorescence spectra of L-PDI dissolved in ethyl acetate. (**a**) 30 mg/L, (**b**) 15 mg/L, (**c**) 7.5 mg/L, (**d**) 3.75 mg/L, (**e**) 1.87 mg/L, (**f**) 0.94 mg/L.

**Figure 6:**
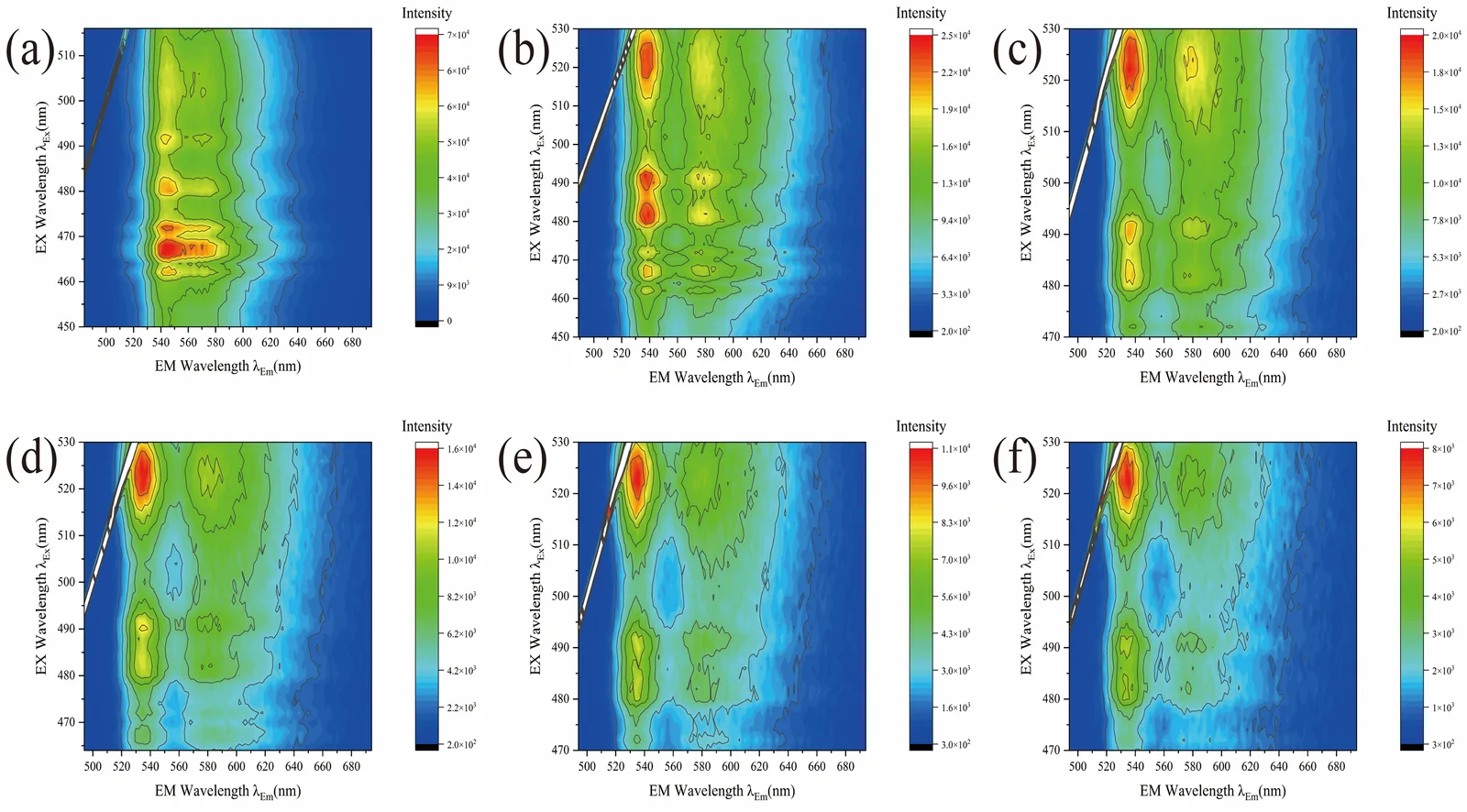
Concentration-dependent 3DEEM fluorescence spectra of L-PDI dissolved in DMSO. (**a**) 30 mg/L, (**b**) 15 mg/L, (**c**) 7.5 mg/L, (**d**) 3.75 mg/L, (**e**) 1.87 mg/L, (**f**) 0.94 mg/L.

Figure 5 presents the concentration-dependent 3DEEM fluorescence spectra of L-PDI dissolved in ethyl acetate. As can be seen, over the concentration range of 0.94–30 mg/L, the positions of the strongest (Ex/Em = 516 nm/526 nm) and the second strongest (Ex/Em = 482 nm/526 nm) emission features remain essentially unchanged, and two distinct emission wavelengths (526 and 564 nm) are observed, indicating that the emitting species remain constant as the concentration varies within the above range. Figure 6 shows the concentration-dependent 3DEEM fluorescence spectra of L-PDI dissolved in DMSO. As shown in the figure, within the concentration range of 30–0.94 mg/L, three excitation wavelengths exhibit relatively high efficiency, namely Ex = 468 nm, 490 nm, and 522 nm. A concentration of 15 mg/L clearly serves as a demarcation point above this concentration, the shorter-wavelength excitation at Ex = 468 nm yields higher emission intensity, with the strongest and second-strongest emission peaks observed at (Ex/Em = 468 nm/544 nm) and (Ex/Em = 472 nm/544 nm), respectively. Below 15 mg/L, the longer-wavelength excitation at Ex = 522 nm dominates, with the strongest and second-strongest emission features located at (Ex/Em = 522 nm/536 nm) and (Ex/Em = 490 nm/536 nm). At the threshold concentration of 15 mg/L, the long- and short-wavelength excitations produce nearly equivalent emission intensities, giving rise to three comparable emission peaks at (Ex/Em = 482 nm/537 nm), (Ex/Em = 492 nm/537 nm), and (Ex/Em = 522 nm/537 nm). This observation suggests that upon dilution from high to low concentration, the energy gap between the ground state and the first excited state undergoes alteration, likely indicating a change in the emitting species.

Based on the strongest emission features in Figure 5 and Figure 6 and Figure S4 shows the concentration-dependent maximum emission intensity of L-PDI in ethyl acetate and DMSO. In the low-concentration range, the intensity increased slowly with concentration, as reflected by the small slope. This behavior arose from the concentration effect with invariant emitting species, where the system likely contained monomers and excimers formed between photoexcited and ground-state monomers. Further concentration increases generated more excimers with enhanced emission. At higher concentrations, trimers and higher aggregates emerged, and their packing modes were solvent-dependent, forming H- and J-aggregates. The significant enhancement of the slope of the concentration-dependent emission intensity curve upon increasing concentration was indicative of an AIE effect.

To further confirm the existence of AIE behavior at high concentrations, the concentration of L-PDI was increased by 100-fold to 1000 mg/L, and the PLQY of the concentrated PDI solution was measured. The experimental results are summarized in Table 3 and Figure S5. It can be clearly observed that when poor solvents such as acetone, ethyl acetate, methanol, and acetonitrile were employed, the PLQY of the concentrated L-PDI solution exhibited an increase of at least 10% compared with that of the dilute solution. In contrast, in good solvents such as pyridine, DMF, and DMSO, the concentrated PDI solution showed a decrease in PLQY of at least 9%. These results demonstrate that L-PDI exhibits pronounced AIE behavior in poor solvents.

**Figure 7:**
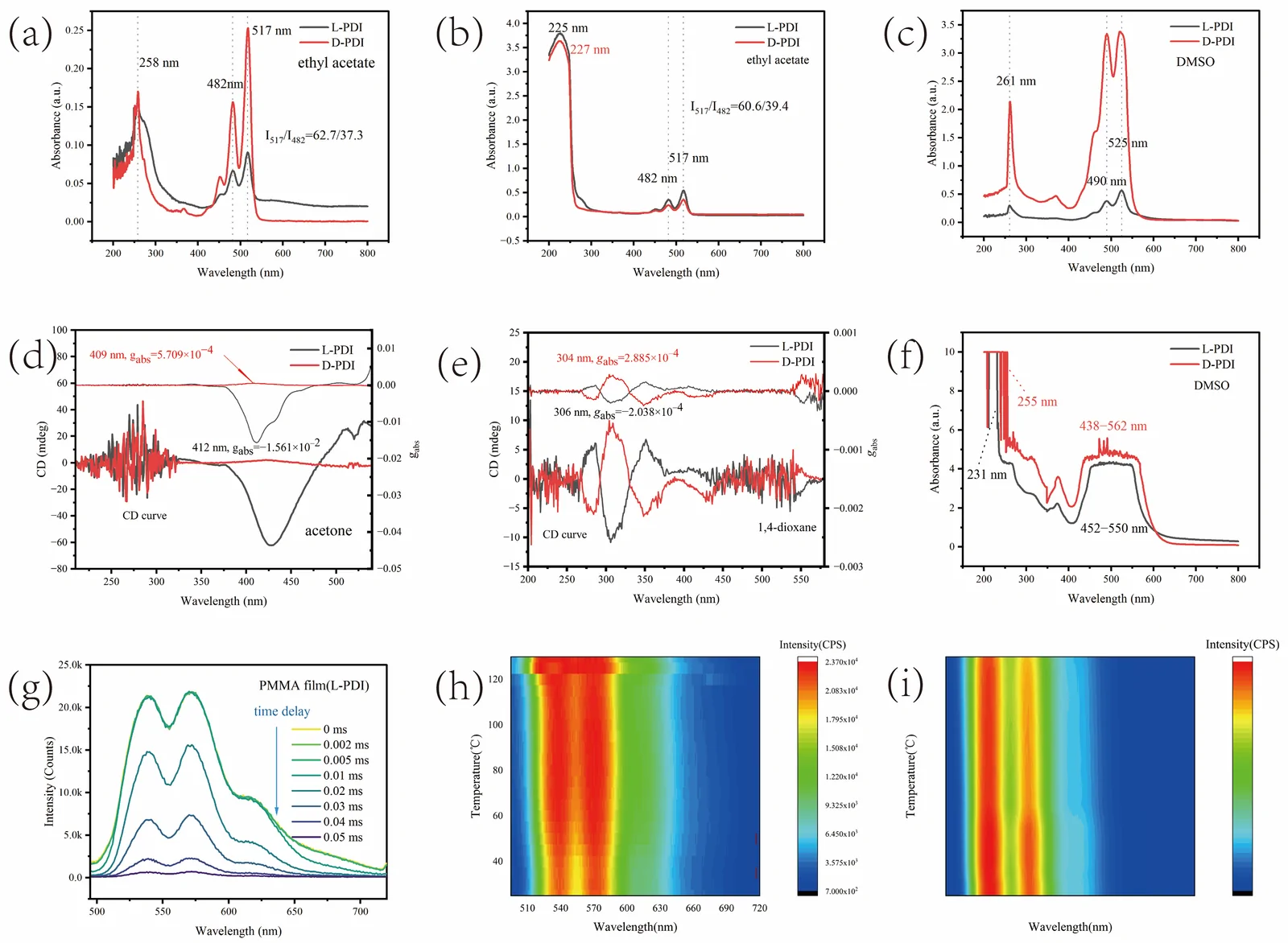
UV-Vis absorption of (**a**,**b**) ethyl acetate and (**c**,**f**) DMSO in dilute ((**a**,**c**) 10 mg/L) and concentrated ((**b**,**f**) 1000 mg/L) solutions, (**d**,**e**) CD spectra of chiral L/D-PDI in acetone and 1,4-dioxane solvents (10 mg/L); (**g**) time-resolved emission spectroscopy, temperature-dependent (**h**) phosphorescence and (**i**) fluorescence spectra of L-PDI in PMMA composite film. Solution concentration is 1000 mg/L for (**g**–**i**).

To investigate the role of chiral alanine in the photoluminescence behavior, we conducted a comparative study on the optical properties of L-PDI and D-PDI. Figure 7a–d present the UV-vis absorption and CD spectra of L-PDI and D-PDI in two different solvents. In the poor solvent ethyl acetate (Figure 7a,b), both L-PDI and D-PDI exhibit sharp, well-resolved triple-peak features in the long-wavelength visible region, with the maximum absorption maxima perfectly superimposed and showing no shift in absorption wavelength. Specifically, the UV-vis absorption spectra of L-DI and D-PDI in the visible region display three distinct peaks at 517, 482, and 454 nm, which are characteristic of the perylene chromophore. The most intense absorption at 517 nm is assigned to the electronic origin (0–0 transition) of the S_0_→S_1_ transition, while the two successive vibronic shoulders at 482 and 454 nm, with energy spacings of approximately 1405 and 2684 cm^−1^ from the 0–0 band, respectively, are attributed to the coupling of the electronic transition with the C=C stretching vibrational modes of the perylene backbone. At a dilute concentration of 10 mg/L, the intensity ratio I_517_/I_482_ is 62.7:37.3, whereas at a concentrated solution of 1000 mg/L, this ratio decreases to 60.6:39.4. The pronounced suppression of the S_0_→S_1_ origin peak at higher concentrations suggests the presence of H-aggregates in the system. Given the substantially higher PLQY observed at elevated concentrations, it is reasonable to infer that multiple aggregation species, including both H- and J-aggregates, coexist under concentrated conditions. For the good solvent DMSO (Figure 7c,f), the fine structure of the absorption bands in the visible region disappears at high concentrations, giving rise to a broad and flattened absorption profile. This observation suggests that the system does not simply consist of discrete H- and J-aggregates; rather, it involves a coexistence of multiple aggregated states with continuous energy distributions and a high degree of disorder. Such a highly disordered configuration precludes the occurrence of AIE.

As shown in Figure 7d, L-PDI exhibits a stronger CD effect than D-PDI in acetone. The maximum value of absorption asymmetry factor (*g*_abs_) for L-PDI is −1.561 × 10^−2^ (at 412 nm), which is significantly larger than that of D-PDI (5.709 × 10^−4^ at 409 nm). This indicates that the high-dielectric-constant solvent acetone selectively stabilizes the twisted conformations of the L-isomer, such as the offset face-to-face π-π stacking excimer (H-type) and the offset head-to-tail π-π stacking excimer (J-type). Consequently, L-PDI exhibits a higher PLQY in acetone. In 1,4-dioxane (Figure 7e), L-PDI and D-PDI show symmetric positive and negative Cotton effects with nearly identical intensities, indicating that 1,4-dioxane does not differentiate in the chiral transmission or retention between the L- and D-α-alanine enantiomers. The variation trend of *g*_abs_ in Figure 7e is consistent with that of the CD spectra. The maximum *g*_abs_ of L-PDI (−2.038 × 10^−4^ at 306 nm) and that of D-PDI (2.885 × 10^−4^ at 304 nm) are comparable in magnitude, suggesting that the low-dielectric-constant solvent 1,4-dioxane exerts weak solvation effects and offers no selective chiral stabilization, which accounts for the observed lower PLQY.

Figure 7g presents the time-resolved emission spectroscopy (TRES) of L-PDI embedded in a PMMA film. As the delay time increases, the emission intensity gradually decreases, while the spectral profile remains essentially unchanged, indicating that the emission wavelength is stable and no color variation occurs over the course of the luminescence decay. The results shown in Figure 7h demonstrate that the fluorescence of L-PDI immobilized in the PMMA film exhibits good thermal stability. Over the temperature range from room temperature to 130 °C, the intensity of the emission peak centered at 537 nm shows little variation. In contrast, the intensity of the 571 nm peak gradually decreases with increasing temperature, which can be attributed to thermal quenching. Notably, the phosphorescence intensity of L-PDI (Figure 7i) displays a significant enhancement for both emission peaks as the temperature increases. This phenomenon suggests the emergence of thermally activated delayed fluorescence (TADF). The superposition of this long-lived TADF (triplet-singlet energy transfer) [38] leads to an overall enhancement of the total emission intensity detected in phosphorescence mode at elevated temperatures.

### 2.3. Aggregation Behavior of PDI-Ala

**Figure 8:**
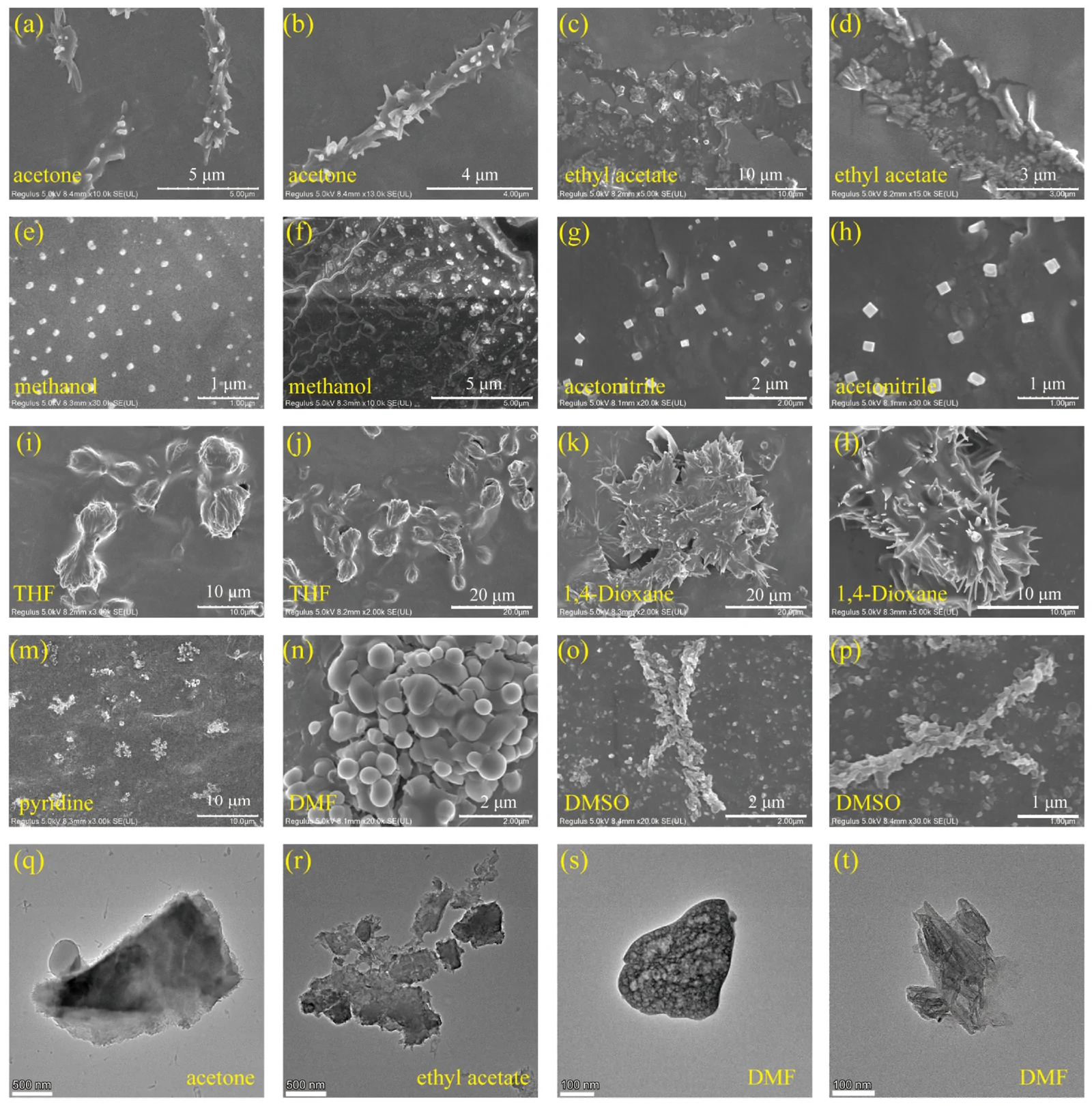
(**a**–**p**) SEM and (**q**–**t**) TEM images of L-PDI dispersed in various solvents.

In order to evaluate the dispersion state of L-PDI in dilute solutions, L-PDI was ultrasonically dispersed in different solvents to prepare SEM/TEM specimens for morphological analysis (detailed preparation methods are provided in SM Section S6). Figure 8a–p presents SEM images of L-PDI dispersed in various solvents, revealing diverse aggregation morphologies influenced by solvent polarity, the solubility and concentration of L-PDI, and the dispersion method. In acetone, ethyl acetate, and DMSO, the aggregates predominantly exhibit dendritic or fibrous structures. In methanol and acetonitrile, sheet-like morphologies are primarily observed, with a small number of vine-like fibers also present in methanol. Conversely, spherical dispersions are prevalent in DMF, while sea-urchin-like aggregates are evident in 1,4-dioxane. TEM analysis in Figure 8q–t indicates that the dimensions of well-dispersed aggregates predominantly fall within the 100–500 nm range. Based on these observations, we propose that the primary molecular packing modes of L-PDI in solvents are H-aggregates and J-aggregates, with a minor contribution from T-aggregates. The variation in packing modes arises from a synergy of weak intermolecular forces, such as π-π interactions (arising from delocalized π-electron clouds on the conjugated aromatic rings), hydrogen bonding, and CH–π interactions. Consequently, the assembly is dictated not by a single dominant force but by the collective interplay of electrostatic forces, van der Waals forces, and the hydrophobic effect.

**Figure 9:**
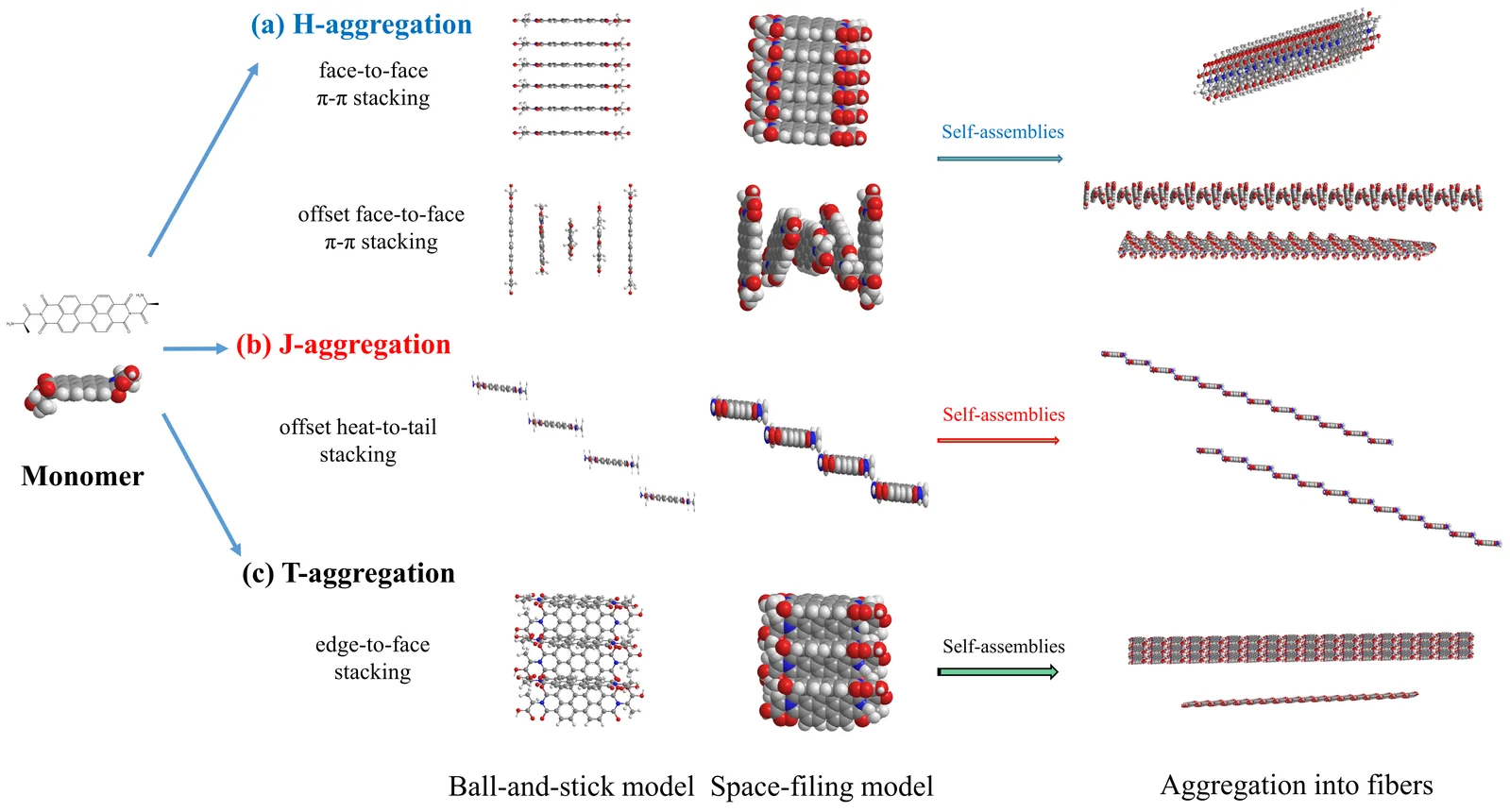
Packing mode of L-PDI dispersed in various solvents.

Figure 9 provides schematic ball-and-stick and space-filling models of the different aggregation states. (a) H-aggregate: This packing mode is driven by π-π interactions between the planar molecular structures, encompassing both the sandwich conformation formed by face-to-face π-π stacking and the long-axis displaced sandwich conformation resulting from offset face-to-face π-π stacking (see Figure 9a). (b) J-aggregate: This packing mode is based on hydrogen bonding (or σ-π attractive interactions) between molecules. Figure 9b illustrates the classic, ideal model of offset head-to-tail stacking. This arrangement effectively minimizes repulsion between π-electron clouds and, compared to H-aggregates, represents a thermodynamically controlled packing state. (c) T-aggregate: This packing mode, also known as edge-to-face stacking, arises from CH–π (or *σ*-π) interactions. The CH–π interaction is a weak force involving a C–H bond (acting as a donor) interacting with an aromatic π-system (acting as an acceptor). Its nature can be considered either a specific type of hydrogen bond (where the hydrogen donor is the hydrogen of C–H bond) or a type of polar-π interaction. This interaction typically manifests when a C–H bond from one molecule (or from a different part of the same molecule) approaches the face of an aromatic ring, leading to the characteristic T-shaped packing configuration.

Among these three interaction types, hydrogen bonding generally exhibits the highest individual bond strength. While π-π interactions are individually weaker than hydrogen bonds, their cumulative effect across multiple points in polyaromatic systems can be substantial. The characteristic face-to-face π-π stacking distance between aromatic rings is approximately 3.3–3.8 Å, with stacking energies on the order of several kcal/mol per aromatic ring pair. CH–π interactions are even weaker than conventional hydrogen bonds, with bond energies typically around 1–3 kcal/mol. However, their collective influence can be significant when multiple such interactions occur within a molecule. Based on the discussion above, and in conjunction with the FT-IR and XRD results presented in Figure 3, it can be inferred that the initial aggregation of L-PDI dispersed in solvents with different polarities is primarily driven by the formation of H- and J-aggregates, which subsequently self-assemble into various fibrous structures.

**Figure 10:**
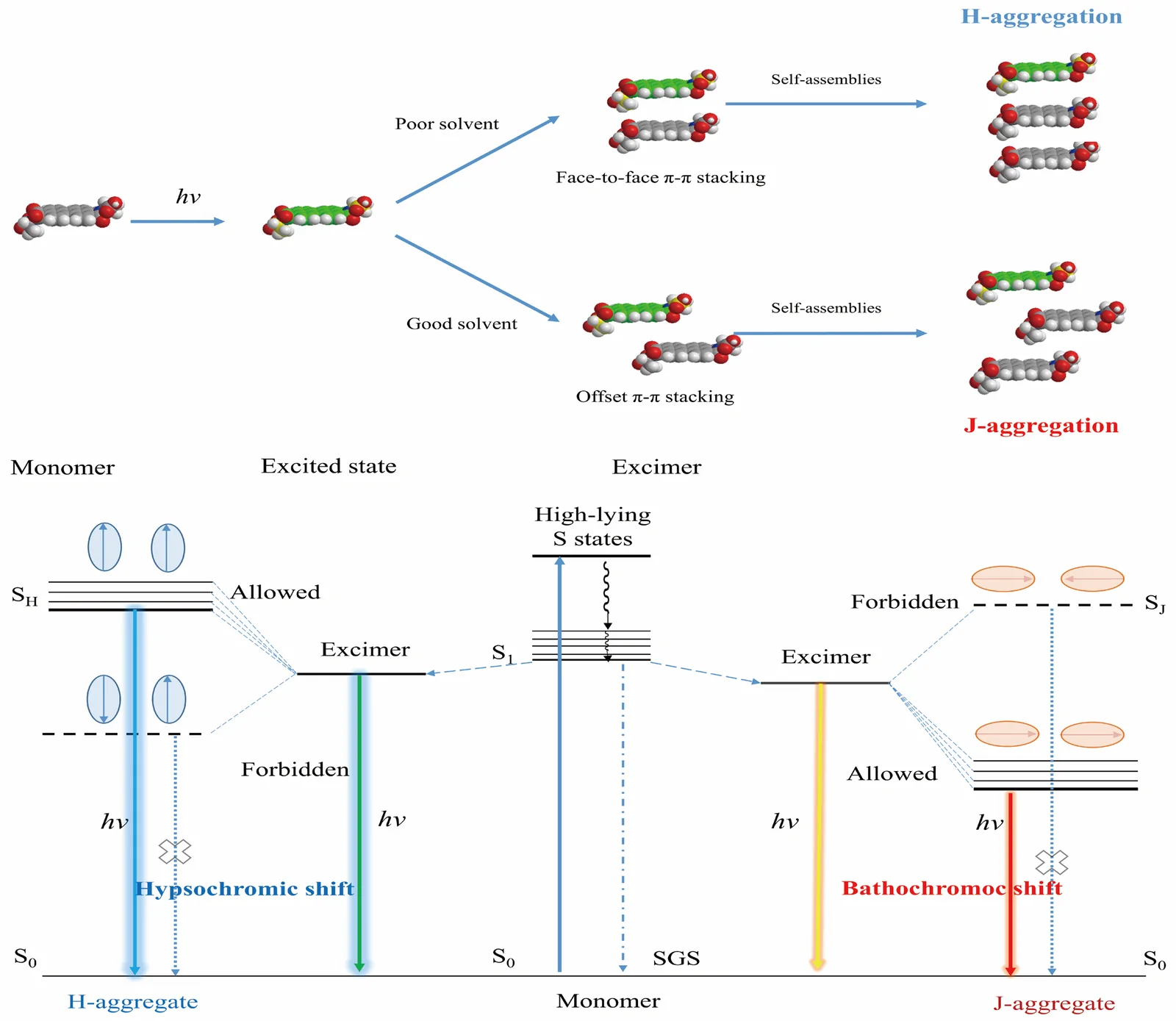
Schematic illustration of the photoluminescence mechanism of L-PDI in different polar solvents.

Based on a comprehensive analysis of the photoluminescence behavior and solvent-dependent aggregation of L-PDI, the proposed photoluminescence mechanism for chiral alanine-functionalized L-PDI is summarized in Figure 10 and elaborated below: (1) In poor solvents, solvated L-PDI (or D-PDI) monomer molecules absorb excitation light at specific wavelengths to form unstable monomeric excited states. The monomeric excited states and ground-state molecules associate to form emissive excimers with face-to-face π-π stacking and offset face-to-face π-π stacking configurations. Among these two types of H-type excimers, the face-to-face π-π stacking excimer exhibits very low luminescence efficiency, whereas the offset face-to-face *π*-*π* stacking excimer serves as the primary emissive species, showing a pronounced blue shift relative to the monomer emission. In addition, offset head-to-tail π-π stacking excimers (J-type) are also formed. Furthermore, the emissive excimers undergo self-assembly in poor solvents to form aggregates of varying sizes (Figure 9), which may consist of trimers or multi-molecule exciplexes. It is noteworthy that the monomer emission is extremely weak, which is attributed to the presence of freely rotatable and vibrating C–C single bonds in the chiral alanine moiety at the diimide position of the monomer molecules. Such bonds enable the monomer to rapidly convert the photon energy of absorbed excitation into kinetic energy of molecular rotation and vibration, leading to non-radiative transitions. However, upon formation of trimers or multi-molecule exciplexes, the absorbed photon energy can be exchanged and transferred among the adjacent monomeric excited-state units and ground-state units that constitute the multi-molecule aggregates, effectively suppressing non-radiative transitions and significantly enhancing the PLQY. (2) In good solvents, a greater number of solvated monomers exist, accompanied by both H-type and J-type excimers, as well as trimers and multi-molecule exciplexes formed through further self-assembly. Owing to the presence of N atoms (with lone-pair electrons) in pyridine and DMF, and O atoms in DMSO, all of which exhibit high electron cloud density, these solvents can act as hydrogen bond acceptors and form stronger intermolecular hydrogen bonds with L-PDI, leading to the formation of more T-aggregates. This results in greater diversity and disorder of the aggregates. Moreover, due to the higher solubility, the aggregates formed in saturated solutions have larger particle sizes, which consequently induces ACQ and reduces the PLQY.

## 3. Experimental Section

### 3.1. Chemicals

Perylene-3,4,9,10-tetracarboxylic dianhydride (PDA, 98%), L-α-alanine (99%), D-α-alanine (98%), β-alanine (99%), imidazole, methanol, acetonitrile, N,N-dimethylformamide (DMF), dimethyl sulfoxide (DMSO), pyridine, acetone, 1,4-dioxane, tetrahydrofuran (THF), and ethyl acetate were all of analytical grade and purchased from Shanghai Macklin Biochemical Co., Ltd. (Shanghai, China). Absolute ethanol and concentrated hydrochloric acid (HCl, 38 wt%, ~12 mol/L) were obtained from Shanghai Titan Scientific Co., Ltd. (Shanghai, China). Deionized water was used throughout the experiments.

### 3.2. Design and Synthesis of PDI-Ala

**Scheme 1:**
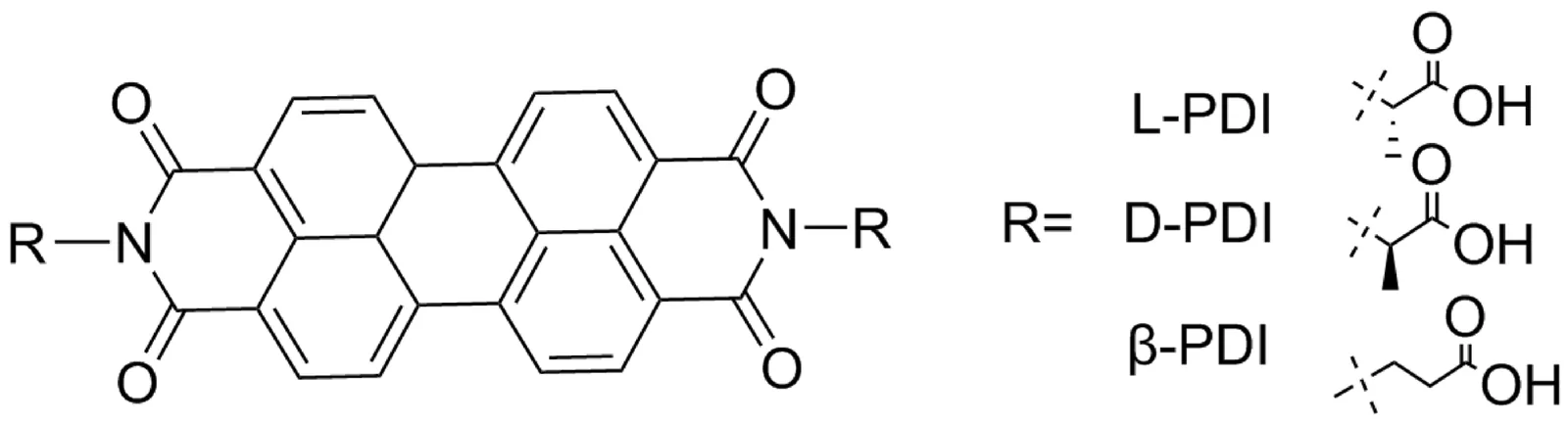
Molecular structures of chiral and achiral PDI-Ala compounds.

Symmetrical PDI-Ala derivatives were synthesized in one step from PDA and the corresponding alanine isomers (Scheme 1). PDA (3.5 mmol), the alanine isomer (D-α-alanine, L-α-alanine, or β-alanine; 28.0 mmol), and imidazole (18.0 g) were placed in a three-necked flask equipped with magnetic stirring. The mixture was stirred at 100 °C for 4 h under argon. After cooling to room temperature, absolute ethanol (100 mL) was added, and the mixture was acidified to pH 2–3 with 2.0 mol/L HCl to induce precipitation. The precipitate was collected by centrifugation (8000 rpm, 5 min), washed thoroughly with deionized water five times, and dried under vacuum at 60 °C to afford PDI-D-α-alanine, PDI-L-α-alanine, and PDI-β-alanine, denoted D-PDI, L-PDI, and β-PDI, respectively.

The structures of the products were verified by ^1^H NMR and ^13^C NMR spectroscopy. L-PDI (1.6125 g, 86.2%): ^1^H NMR (600 MHz, DMSO-*d*_6_) *δ* 12.99 (s, 2H), 7.20–7.92 (m, 8H), 5.52 (s, 2H), 1.44 (m, 6H). ^13^C NMR (150 MHz, DMSO-*d*_6_) *δ* 170.42, 161.34, 132.99, 130.46, 127.04, 124.36, 121.23, 120.56, 48.68, 15.49. IR(solid): *ν* = 3437($\nu_{O-H}$), 1745($\nu_{s(C-O)})$, 1697($\nu_{C=O}$), 1645($\nu_{C=C}$), 1585($\nu_{as(C-O)}$), 1439, 1367($\nu_{C-N}$), 1256, 1188, 1100 cm^−1^. L-PDI (1.5994 g, 85.8%): ^1^H NMR (600 MHz, DMSO-*d*_6_) *δ* 12.98 (s, 2H), 7.20–7.92 (m, 8H), 5.52 (s, 2H), 1.45 (m, 6H). ^13^C NMR (150 MHz, DMSO-*d*_6_) *δ* 170.42, 161.34, 132.99, 130.46, 127.04, 124.35, 121.23, 120.56, 48.68, 15.50. β-PDI(1.5657 g, 83.7%): ^1^H NMR (600 MHz, DMSO-*d*_6_) *δ* 11.90 (s, 2H), 7.90(d, 4H), 7.75 (d, 4H), 4.73 (t t, 4H), 2.65 (m, 4H). ^13^C NMR (150 MHz, DMSO-*d*_6_) *δ* 172.02, 162.13, 133.10, 130.75, 127.63, 124.40, 122.35, 121.84, 39.15, 32.46.

### 3.3. Characterization

^1^H NMR and ^13^C NMR spectra were recorded on a Bruker Ascend 600 MHz spectrometer (Billerica, MA, USA). X-ray diffraction (XRD) patterns were collected on a Rigaku Smart Lab diffractometer (Akishima-shi, Japan) over a 2θ range of 10–80 deg. Fourier transform infrared (FT-IR) spectra were recorded on a Bruker Vector-80 spectrometer in the range of 400–4000 cm^−1^ using KBr pellets. Morphologies were characterized by scanning electron microscopy (SEM, Hitachi Regulus 8100, Tokyo, Japan) and transmission electron microscopy (TEM, FEI Talos F200i S, Hillsboro, OR, USA). UV-vis spectra were recorded on an Agilent Cary 5000 spectrophotometer (Santa Clara, CA, USA). Photoluminescence (PL) and three-dimensional excitation-emission matrix (3DEEM) spectra were obtained using a Horiba FluoroMax-4P fluorescence spectrometer (Edison, NJ, USA) equipped with a 150 W Xe lamp. Photoluminescence quantum yield (PLQY) was measured using an integrating sphere accessory (Quanta-F-3029, HORIBA, Edison, NJ, USA), and CD spectra were recorded on an Applied Photophysics Chirascan instrument (Leatherhead, UK).

## 4. Conclusions

Alanine-functionalized PDI derivatives were successfully synthesized as a model system to investigate the interplay among molecular chirality, solvent environment, supramolecular aggregation, and luminescence behavior. Although alanine improves water compatibility, the planar PDI core still favors strong face-to-face stacking in the solid state and in water, leading to severe fluorescence quenching and ACQ. L-PDI exhibits pronounced solvent polarity, stacking mode, and aggregation state-dependent photoluminescence behaviors, with poor solvents favoring hypsochromic-shifted emission and good solvents inducing bathochromic-shifted emission. Specifically, in poor solvents, a relatively high PLQY is observed. For example, at a concentration as high as 1000 mg/L, the PLQY in ethyl acetate reaches 98.5%, which is 1.68 times that at a dilute concentration of 10 mg/L (58.3%). This is because two types of H-aggregates coexist in poor solvents: face-to-face π-π stacking and offset face-to-face π-π stacking (Figure 9). The latter adopts a herringbone-like packing mode, which facilitates electron and hole transport and thus enables higher emission efficiency [39]. In addition, due to the limited solubility in poor solvents, a large number of H-excimers (dimers) are present. In good solvents, however, the higher solubility leads to increased aggregation degrees of the aggregates, which induces ACQ, resulting in a relatively low PLQY at high concentrations. For instance, in DMSO and DMF solutions at a concentration of 1000 mg/L, the PLQY of L-PDI drops to as low as 13.8% and 24.0%, respectively.

Spectroscopic, structural, and microscopic results indicate that the luminescence differences in L-PDI in these solvents mainly arise from solvent-regulated aggregation structures [40,41], restricted dynamic intramolecular rotation, and the types of *π*-*π* interactions, accompanied by diverse self-assembled morphologies. Overall, the optical response of PDI-Ala is governed by the formation of excimers/exciplexes, together with the balance between H-type and J-type aggregates of varying morphologies and sizes. This work deepens the understanding of aggregation-controlled emission in chiral PDI systems and guides the design of chiroptical, self-assembled, and stimuli-responsive luminescent materials.

## Figures and Tables

**Table 1:** Photoluminescence spectral characteristics and properties of L-PDI in different solvents.

Solvent	$\lambda_{ex}$ (nm)	$\lambda_{em}$(*i*) (nm) (Bimodal)	Integral Interval (nm)	Integral Area	PL QY (%)	Lifetimes (Ratios) *τ*_1_, *τ*_2_ (ns)
acetone	482	526 *, 567	500–750	6.6136 × 10^6^	75.8 ± 0.3	7.80 (96.13%) 93.21 (3.87%)
ethyl acetate	482	525 *, 562	500–750	3.1667 × 10^6^	58.3 ± 0.8	5.45 (99.73%) 260.92 (0.27%)
methanol	482	537 *, 570	500–750	2.0517 × 10^7^	69.3 ± 0.2	
acetonitrile	482	523 *, 566	500–750	7.4665 × 10^6^	81.9 ± 4.4	
THF	468	533 *, 568	500–750	2.5169 × 10^7^	79.2 ± 0.7	
1,4-dioxane	416	536 *, 568	500–750	2.2628 × 10^6^	79.1 ± 1.3	
pyridine	416	545 *, 577	500–750	1.2821 × 10^6^	39.9 ± 0.6	6.79 (99.26%) 48.07 (0.74%)
DMF	482	538 *, 571	500–750	1.1446 × 10^7^	46.2 ± 0.1	
DMSO	482	538 *, 590	500–750	1.2941 × 10^6^	18.2 ± 0.5	

Solution concentration: 10 mg/L. * The major peak of the doublet.

**Table 2:** Solvatochromic parameters of various solvents.

Solvent	Dielectric Constant ε	Polarity Index	Hildebrand Solubility Parameters (MPa^1/2^)	Number of Hydrogen Bond Acceptors	Number of Hydrogen Bond Donors	Number of Terminal Methyl Groups	Total Numbers
acetone	21	5.1	20.0	1	0	2	3
ethyl acetate	6	4.4	18.2	2	0	2	4
methanol	33	5.1	29.7	1	1	1	3
acetonitrile	36	5.8	24.3	1	0	1	2
THF	7.6	4.0	19.4	1	0	0	1
1,4-dioxane	2.2	4.8	20.5	2	0	0	2
pyridine	12.5	5.3	17.3	1	0	0	1
DMF	37	6.4	24.8	1	0	0	1
DMSO	47	7.2	26.6	1	0	0	1

**Table 3:** PLQY of L-PDI in dilute and concentrated solutions.

	PLQY(%)		
Solvent	Dilute Solution	Concentrated Solution	Relative Increase (%)
	10 mg/L	1000 mg/L	
acetone	75.8 ± 0.3	88.1 ± 1.2	16.2
ethyl acetate	58.3 ± 0.8	98.5 ± 0.5	70.0
methanol	69.3 ± 0.2	86.4 ± 0.3	24.7
acetonitrile	81.9 ± 4.4	90.2 ± 2.6	10.1
THF	79.2 ± 0.7	78.5 ± 0.9	−0.8
1,4-dioxane	79.1 ± 1.3	82.1 ± 1.1	3.8
pyridine	39.9 ± 0.6	36.3 ± 0.7	−9.0
DMF	46.2 ± 0.1	24.0 ± 0.3	−48.1
DMSO	18.2 ± 0.5	13.8 ± 0.6	−24.2

## Data Availability

Data will be made available on request.

## Supplementary Materials

The following supporting information can be downloaded at: https://www.mdpi.com/article/10.3390/molecules31173102/s1. Figure S1. CIE chromaticity coordinates of L-PDI emission in different polar solvents. Table S1. CIE(x,y) of L-PDI emission in different polar solvents. Table S2. d-spacings of PDI-Ala in solid and PMMA film. Figure S2. PLQYs of L-PDI in different solvents. Solution concentration: 10 mg/L. Figure S3. XRD local magnification spectra of PDI-Ala and PMMA composite films. Figure S4. Concentration-dependent PL intensity of L-PDI in ethyl acetate and DMSO. Figure S5. PLQYs of L-PDI in concentrated solution. Concentration: 1000 mg/L.
