# Supplementary material for: Chiral Alanine Functionalized Perylene Diimides: Supramolecular Self-Assembly and Optical Properties
Source: Molecules. 2026 Sep 4;31(17):3102. doi: 10.3390/molecules31173102 (PMC13567569; doi:10.3390/molecules31173102)
Supplement: Supplementary file 1 [file molecules-31-03102-s001.zip › molecules-4529442-supplementary.pdf]

Supplementary Material

Chiral Alanine Functionalized Perylene Diimides: Supramolecular Self-Assembly and Optical Properties

Di Fan [1], Fan Qi [2,*], Nan Li [3,4], Rongli Liu [2], Haitao Ren [5] and Guodong Fan [2,4,*]

[1] School of Artificial Intelligence, Shaanxi Polytechnic University, Xianyang 712000, China
[2] School of Medicine, Xi'an Peihua University, Xi'an 710125, China
[3] College of Chemistry and Materials Science, Weinan Normal University, Weinan 714099, China
[4] Key Laboratory of Auxiliary Chemistry &Technology for Chemical Industry, Shaanxi University of Science & Technology, Ministry of Education, Xi'an 710021, China
[5] Shaanxi Key Laboratory of Liquid Crystal Polymer Intelligent Display, Technological Institute of Materials & Energy Science (TIMES), Xijing University, Xi'an 710123, China
* Correspondence: qifan@mail.nankai.edu.cn (F.Q.); fangd@sust.edu.cn (G.F.)

S1. CIE chromaticity coordinates of L-PDI

In order to better understand the PL color of samples, a series of color coordinate charts of L-PDI in different polar solvents were drawn according to the emission spectrum of **Figure2**a. The results are shown in **Figure S1** and **Table S1**. In different solvents, the emission of L-PDI exhibited a marked variation, shifting from orange-red to green light. In high-polarity aprotic solvents such as DMSO, pyridine, and DMF, the emission was located in the orange-red region. It appeared as yellow light in 1,4-dioxane and as bright green light in methanol, acetone, acetonitrile, and ethyl acetate. The photoluminescence of L-PDI exhibited a pronounced solvatochromic effect, with the emission color tuning from orange-red in high-polarity solvents such as DMSO (0.5433, 0.4539) to green in solvents like acetonitrile (0.3561, 0.6255), and ethyl acetate (0.3477, 0.6287).

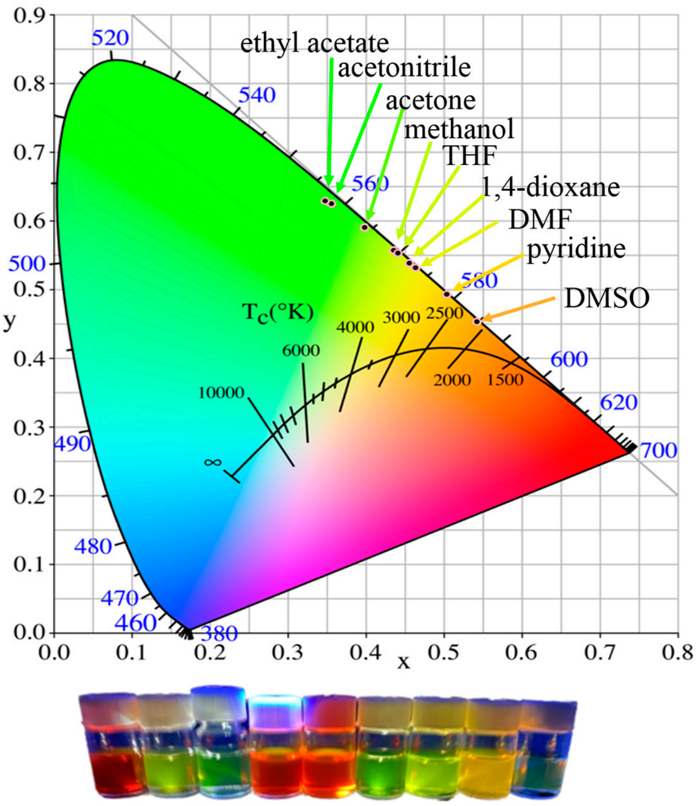

FigureS1. CIE chromaticity coordinates of L-PDI emission in different polar solvents.

(The solvents corresponding to the PL photographs at the bottom, from left to right, are DMSO, methanol, acetonitrile, DMF, pyridine, acetone, 1,4-dioxane, THF, and ethyl acetate, all at a concentration of 10 mg/L)

Table S1. CIE(x,y) of L-PDI emission in different polar solvents

No.	solvents	CIE	
		x	y
1	DMSO	0.5433	0.4539
2	methanol	0.4364	0.5570
3	acetonitrile	0.3561	0.6255
4	DMF	0.4638	0.5319
5	pyridine	0.5042	0.4927
6	acetone	0.3980	0.5904
7	1,4-dioxane	0.4566	0.5393
8	THF	0.4412	0.5533
9	ethyl acetate	0.3477	0.6287

Solution concentration: 10 mg/L.

S2. PLQYs of chiral PDI-Ala in different solvents

To further investigate the photoluminescence efficiency of L-PDI in different solvents, the PLQYs of different solutions were measured using an integrating sphere, and the test results are presented in **Figure S2**.

S3. XRD patterns of PDI-Ala and PMMA composite film

Figure S3 and **Table S2** provide the intermolecular d-spacings of all PDI-Ala in the solid state and in PMMA films, and the corresponding packing modes are marked.

Table S2. d-spacings of PDI-Ala in solid and PMMA film

compound	d_1-spacings (Å)	d_2-spacings (Å)	d_3-spacings (Å)	d_4-spacings (Å)
β-PDI(Solid)	3.41[a]	3.22[b]	3.04[b]	/
D-PDI(Solid)	3.47[a]	3.37[b]	3.26[b]	/
L-PDI(Solid)	3.46[a]	3.36[b]	3.25[b]	/
L-PDI(PMMA film)	3.72[c]	3.36[b]	3.34[b]	4.13[d]

[a] represents molecular spacing of face-to-face π-π stacking, [b] represents molecular spacing of offset face-to-face π-π stacking, [c,d] represent molecular spacing of dimers, excimers or exciplexes.

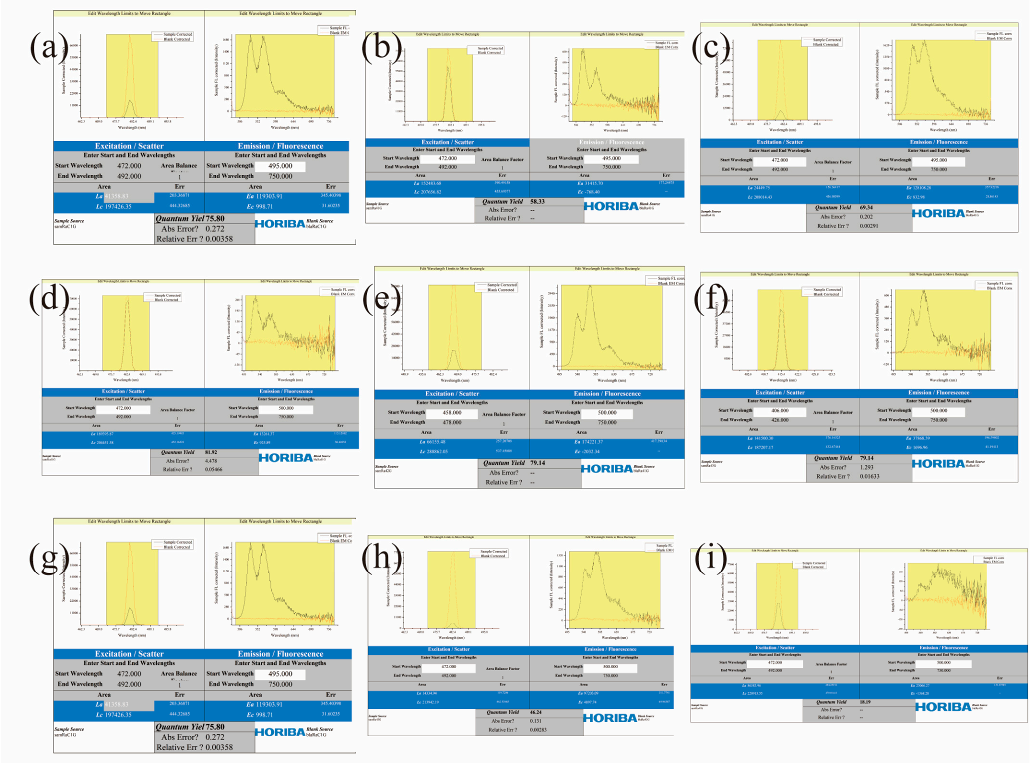

(a) acetone, (b) ethyl acetate, (c) methanol, (d) acetonitrile, (e) THF, (f) 1,4-dioxane,

(g) pyridine, (h)DMF, (i) DMSO.

Figure S2. PLQYs of L-PDI in different solvents. Solution concentration: 10 mg/L.

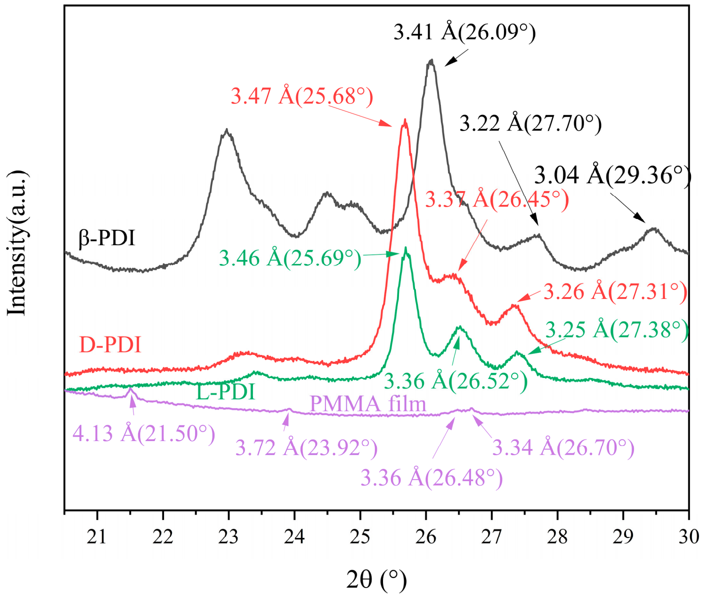

Figure S3. XRD local magnification spectra of PDI-Ala and PMMA composite films

S4. Concentration-dependent PL intensity of L-PDI in ethyl acetate and DMSO

Based on the strongest emission features in **Figures 5,6**, **Figure S4** presents the concentration-dependent maximum emission intensity of L-PDI in ethyl acetate and DMSO.

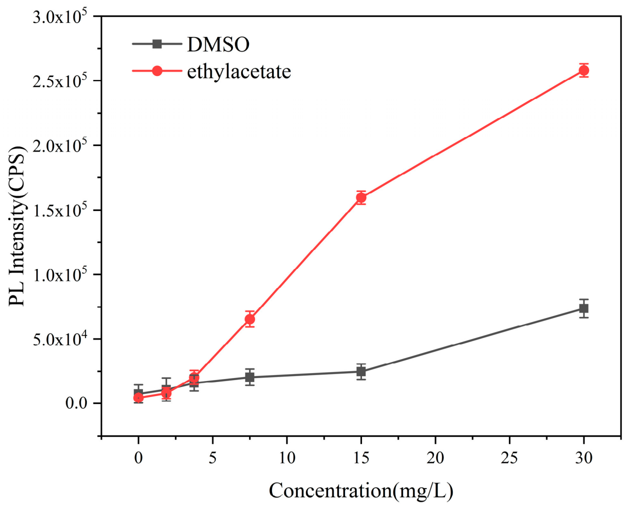

Figure S4. Concentration-dependent PL intensity of L-PDI in ethyl acetate and DMSO

S5. PLQY of L-PDI in concentrated solutions

To further confirm the existence of AIE behavior at high concentrations, the concentration of L-PDI was increased by 100-fold to 1000 mg/L , and the PLQY of the concentrated PDI solution was measured. The solution was prepared as follows: 50 mg of L-PDI was dissolved in 50 mL of solvent, followed by magnetic stirring and ultrasonication to maximize dispersion. The solution was then allowed to stand for 30 min, and the upper layer was sampled for measurement to avoid the inclusion of non-uniform suspensions or solid particulates. Owing to solubility limitations, solid precipitates were observed at the bottom of the L-PDI solution in most solvents, indicating that the actual concentration corresponded to a saturated solution rather than the nominal 1000 mg/L. The experimental results are shown in **Figure S5.**

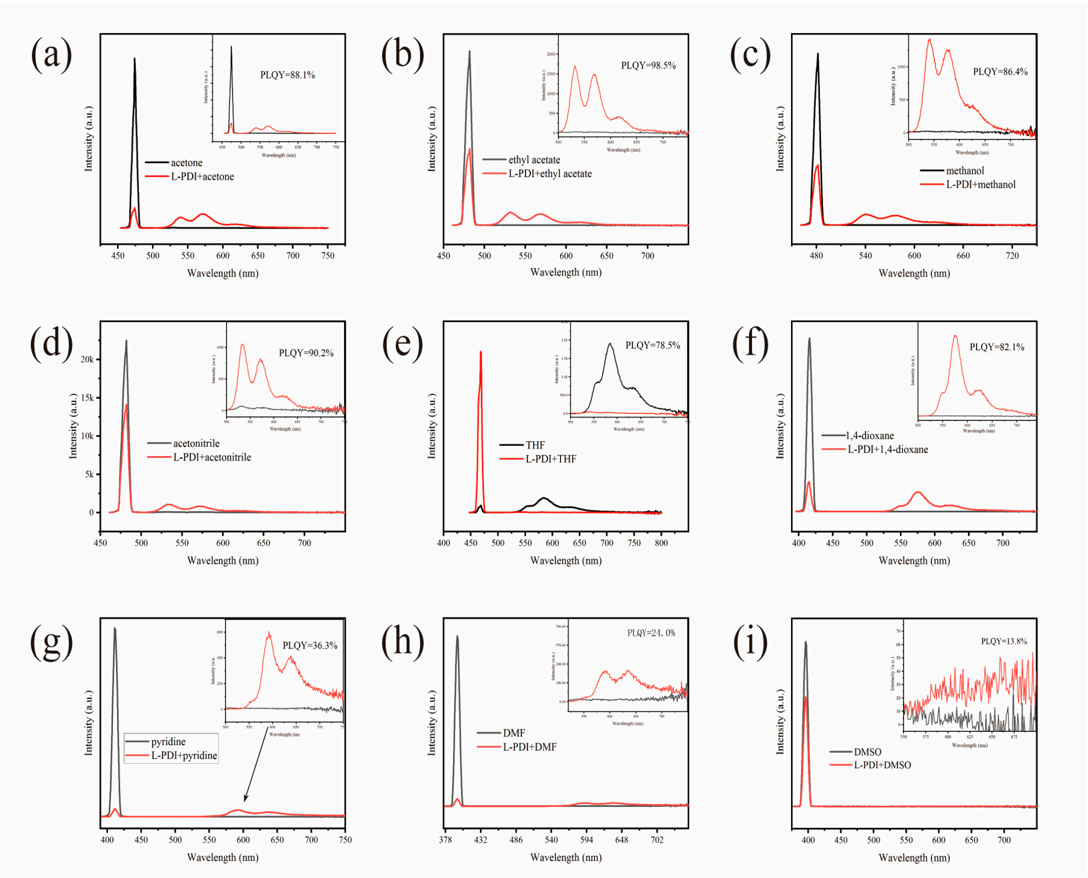

(a) acetone, (b) ethyl acetate, (c) methanol, (d) acetonitrile, (e) THF, (f) 1,4-dioxane, (g) pyridine, (h)DMF, (i) DMSO.

Figure S5. PLQYs of L-PDI in concentrated solution. Concentration: 1000 mg/L.

S6. Method for the preparation of SEM/TEM samples

SEM samples were prepared via the drop-casting method. A small amount of PDI powder was added to different solvents and ultrasonically dispersed to form highly dilute transparent solutions (solution concentration 10 mg/L). A small volume of each solution was then drop-cast onto aluminum foil attached to the sample holder using a pipette. After air drying, the samples were sputter-coated with gold prior to imaging.

TEM samples were prepared by ultrasonically dispersing the powder samples in different solvents for about 10 min to obtain a homogeneous suspension(solution concentration 10 mg/L). A drop of the suspension was then placed onto a carbon-coated copper grid and dried under ambient conditions before measurement.
